# Transformation of Human Urothelial Cells (UROtsa) by As^3+^ and Cd^2+^ Induces the Expression of Keratin 6a

**DOI:** 10.1289/ehp.10279

**Published:** 2007-12-17

**Authors:** Seema Somji, Chandra S. Bathula, Xu Dong Zhou, Mary Ann Sens, Donald A. Sens, Scott H. Garrett

**Affiliations:** Department of Pathology, School of Medicine and Health Sciences, University of North Dakota, Grand Forks, North Dakota, USA

**Keywords:** arsenic, bladder cancer, cadmium, keratin, urothelium, UROtsa

## Abstract

**Background:**

Cadmium and arsenite can directly and malignantly transform the UROtsa cell line. The tumor heterotransplants produced from these transformed cells have histologic features consistent with human bladder cancer. Previous microarray analysis of total RNA from the parental and transformed cells suggested that keratin 6a was overexpressed as a result of cell transformation.

**Objectives:**

Our goals were to verify overexpression of keratin 6a in Cd^2+^- and As^3+^-transformed UROtsa cells, the corresponding tumor heterotransplants, and human bladder cancer biopsy specimens and to assess what factors may be involved in keratin 6a overexpression.

**Methods:**

Expression was assessed with real-time polymerase chain reaction, Western blot analysis, and immunohistochemistry. We used the effect of addition and deletion of potential growth factors in the cell culture growth medium to assess possible pathways used in keratin 6a overexpression.

**Results:**

Cd^2+^- and As^3+^-transformed cells grown in serum-containing growth medium, as well as the derived tumor heterotransplants, overexpressed keratin 6a mRNA and protein compared with UROtsa cells grown in serum-containing growth medium. Immunostaining of keratin 6a in tumor heterotransplants showed focal staining of the tumor cells that was localized to the cytoplasm. Focal immunostaining of keratin 6a was also found in some but not all archival patient specimens of high-grade bladder cancer, confirming translation of the results to human bladder cancer. Studies on growth factor deletion and addition indicated that the level of keratin 6a expression was regulated by the presence of both insulin and epidermal growth factor (EGF). In contrast, growth factors had no effect on the elevated levels of keratin 6a expression found in transformed UROtsa cells.

**Conclusions:**

Our present studies suggest that keratin 6a expression may be a biomarker for malignant urothelial cells that possess an activated EGF and or insulin growth factor pathway.

This laboratory has demonstrated that both cadmium and arsenite can directly malignantly transform the UROtsa cell line (Sens et al. 2004). It was also shown that the tumor heterotransplants produced by the Cd^2+^- and As^3+^-transformed cells had histologic features consistent with human transitional cell carcinoma of the bladder. The parent UROtsa cell line was developed from a primary cell culture of human urothelium that was immortalized using the SV40 large T-antigen (Petzoldt et al. 1994, 1995). The UROtsa cells proliferate in a simple serum-containing growth medium, retain a normal cytogenetic profile, grow as a contact-inhibited monolayer, and possess an undifferentiated morphology consistent with basal epithelial cells. They are not tumorigenic as judged by their inability to form colonies in soft agar and tumors in nude mice.

Our laboratory also adapted the UROtsa cells to grow in a serum-free growth medium (Rossi et al. 2001). Under serum-free conditions, the cells exhibited enhanced differentiation, as evidenced by the presence of a stratified morphology consistent with the structural features associated with the intermediate layers of the urothelium. The cells grown in serum-free medium retained the properties of immortality, contact inhibition, and nontumorigenicity.

Total RNA isolated from the parental UROtsa cells and their As^3+^- and Cd^2+^-transformed sublines grown in serum-containing growth medium were subjected to microarray analysis employing the U133 Plus 2.0 Affymetric chip. One of the stronger differentially expressed signals between the parental and the As^3+^-transformed cells was the keratin 6a gene. Our first goal in the present study was to confirm the differential expression of keratin 6a in the parental and transformed UROtsa cell lines at both the mRNA and protein levels. The second goal was to determine the expression of the keratin 6a gene in tumor heterotransplants derived from the As^3+^- and Cd^2+^-transformed UROtsa cells. The final aim was to demonstrate that the results from this model system could potentially translate to human disease by determining if keratin 6a was overexpressed in archival specimens of human bladder cancer.

## Materials and Methods

### Cell culture

Stock cultures of the parent UROtsa cell line and the cell lines malignantly transformed with either 1 μM Cd^2+^ or As^3+^ were maintained in 75 cm^2^ tissue culture flasks in either serum or serum-free growth medium as described previously (Rossi et al. 2001; Sens et al. 2004). Briefly, the serum-containing growth medium was Dulbecco’s modified Eagle’s medium (DMEM) containing 5% vol/vol fetal calf serum. The serum-free growth medium was composed of a 1:1 mixture of DMEM and Ham’s F-12 supplemented with selenium (5 ng/mL), insulin (5 μg/mL), transferrin (5 μg/mL), hydrocorti-sone (36 ng/mL), triiodothyronine (4 pg/mL), glutamine (2 mM), and epidermal growth factor (EGF; 10 ng/mL). When confluent, the cells were subcultured at a 1:20 ratio using trypsin–EDTA. We incubated cultures at 37°C in a 5% CO_2_:95% air atmosphere and fed the cultures fresh growth medium every 3 days. The Cd^2+^- and As^3+^-transformed cell lines were serially passaged 10 times in Cd^2+^- and As^3+^-free growth medium before use in any experimental protocols or before heterotrans-plantation into nude mice. The parental cell line is defined by the designation UROtsa. The cells transformed with Cd^2+^ or As^3+^ in serum-containing growth medium are defined by the designations URO-CDSC and URO-ASSC, respectively. The cells transformed with Cd^2+^ or As^3+^ in serum-free growth medium are defined by the designations URO-CDSF and URO-ASSF, respectively.

### Tumor heterotransplants

The production of subcutaneous nude mouse heterotransplants from the Cd^2+^- and As^3+^-transformed UROtsa cell lines has been previously described (Sens et al. 2004). Briefly, 5 × 10^6^ cells supended in 0.2 mL phosphate-buffered saline were injected in the dorsal thoracic mid-line of nude (NCr-*nu/nu*) mice. We injected 10 mice with each transformed isolate. In the present study, we used the tissues taken from these tumors to determine the expression of keratin 6a mRNA and protein. In addition to tumor tissue harvested for histology, we also took samples from these specimens for the preparation of total RNA and protein. Protocols for the use of animals in this study were approved by the University of North Dakota Committee for the Care and Use of Animals, which is in compliance with all current National Institutes of Health (NIH) guidelines and regulations (NIH 2002). The facilities for animal care are accredited by the American Association for Accreditation of Laboratory Animal Care (AAALAC).

### Bladder specimens for the retrospective immunohistochemical analysis of keratin 6a expression

Tissue sections for the immunohistochemical analysis of keratin 6a expression in the human bladder were obtained from archival paraffin blocks that originated from previously completed patient diagnostic procedures. In all cases, the type of bladder cancer was transitional cell carcinoma, which represents the predominant form of bladder cancer in humans. These archival specimens contained no patient identifiers and use was approved by the University of North Dakota Internal Review Board.

### Immunostaining for keratin 6a in tumor heterotransplants and archival human specimens

We routinely fixed tissues in 10% neutral-buffered formalin for 16–18 hr. All tissues were transferred to 70% ethanol and dehydrated in 100% ethanol. Dehydrated tissues were cleared in xylene, infiltrated, and embedded in paraffin. Serial sections were cut at 3–5 μm for use in immunohistochemical protocols. Prior to immunostaining, sections were immersed in preheated Target Retrieval Solution (catalog no. S1699; Dako, Carpinteria, CA) and heated in a steamer for 20 min. The sections were allowed to cool to room temperature and immersed into Tris-buffered saline with Tween-20 for 5 min. Keratin 6a was localized using a monoclonal antibody as the primary antibody (Cat. no. ab2393; Abcam, Cambridge, MA). For archival human specimens, the primary antibody was localized using the Dakocytomation peroxidase conjugated EnVision Dual link system (Cat. no. K4061; Dako). We used liquid diaminobenzidine for visualization (Dakocytomation liquid DAB substrate chromogen system, Cat. no. K3466; Dako). Slides were rinsed in distilled water, dehydrated in graded ethanol, cleared in xylene, and coverslipped. For tumor heterotransplants, the primary antibody was localized using the DakoCytomation ARK (Animal Research Kit; Dako), and peroxidase (Cat. no. K3954; Dako). This system minimizes reactivity of secondary antimouse antibody with endogenous immunoglobulin that may be present in the heterotransplant-generated specimen. Liquid diaminobenzidine (DAB) was used for visualization. Slides were rinsed in distilled water, dehydrated in graded ethanol, cleared in xylene, and coverslipped.

### Real-time analysis of keratin 6a mRNA expression

We assessed the measurement of keratin 6a mRNA expression with real-time reverse transcription polymerase chain reaction (RT-PCR) using commercially available primers (Cat. no. QT 01003541; QIAGEN, Valencia, CA). Total RNA was purified from the cells lines grown in 9.6-cm^2^ culture wells in triplicate, and 1 μg was subjected to cDNA synthesis using the iScript cDNA synthesis kit (Cat. no. 170–8890; Bio-Rad Laboratories, Hercules CA) in a total volume of 20 μL. Real-time RT-PCR was performed using the SYBR Green kit (Cat. no. 170–8890; Bio-Rad Laboratories) with 2 μL cDNA and 0.2 μM primers in a total volume of 20 μL in an iCycler iQ real-time detection system (Bio-Rad Laboratories). Amplification was monitored by SYBR Green fluorescence. Cycling parameters consisted of denaturation at 95°C for 15 sec, annealing at 55°C for 45 sec, and extension at 72°C, which gave optimal amplification efficiency. We determined the levels of keratin 6A mRNA relative to the UROtsa cells grown in serum-containing medium using serial dilutions of this sample as the standard curve. The resulting relative levels were then normalized to the change in glyceraldehyde-3-phosphate dehydrogenase (G3PDH) expression assessed by the same assay using the primers, sense: TCCTCTGACTTCAACAGCGACAC and antisense: CACCCTGTTGCTGTAGC CAAATTC, giving a product size of 126 base pairs and with the cycling parameters of annealing/extension at 62°C for 45 sec and denaturation at 95°C for 15 sec.

### Western blot analysis of keratin 6a expression

Confluent cultures were harvested in 2% sodium dodecyl sulfate (SDS) and 50 mM Tris–HCl, pH 6.8, followed by boiling for 10 min and DNA shearing through a 23-ga needle. We determined protein concentration using the bicinchoninic acid protein assay (Pierce Chemical Co., Rockford, IL) before 100 mM dithiothreitol was added to each sample. Total cellular protein (20 μg) was separated on a 12.5% SDS-polyacrylamide gel electrophoresis gel and transferred to a hybond-P polyvinylidene difluoride membrane (Cat. no. RPN2020F; Amersham Biosciences, Piscataway, NJ). Membranes were blocked in Tris-buffered saline (TBS) containing 0.1% Tween-20 (TBS-T) and 5% (wt/vol) nonfat dry milk for 1 hr at room temperature. After blocking, the membranes were probed with the keratin 6a primary antibody (1:500) (Abcam) in blocking buffer overnight. After washing 3 times in TBS, we incubated the membranes with the antimouse secondary antibody (1:2000) in antibody dilution buffer for 1 hr. The blots were visualized using the Phototope-HRP Western blot detection system (Cat. no. 7071; Cell Signaling Technology, Beverly, MA).

### UROtsa cell culture in the presence or absence of insulin and/or EGF

UROtsa cells were grown to confluency in 6-well plates in DMEM media supplemented with 5% fetal calf serum. At confluence, EGF (10 ng/mL), insulin (5 μg/mL), or insulin + EGF (5 μg/mL and 10 ng/mL, respectively) was added to the serum-containing medium for 16 hr, and the cells were prepared as above for the determination of keratin 6a mRNA and protein. We performed the corollary experiment using serum-free growth medium where EGF, insulin, and EGF + insulin were removed from the medium for 16 hr.

### Statistical analysis

All cell culture experiments were performed in triplicate, and the results are expressed as the mean ± SE. Statistical analyses were performed using Systat software (Systat Software, Inc., Chicago, IL) using separate variance *t*-tests, analysis of variance (ANOVA) with Tukey post-hoc testing. Unless otherwise stated, the level of significance was 0.05.

## Results

### Expression of keratin 6a mRNA and protein in normal and transformed UROtsa cell lines

**Figure 1:**
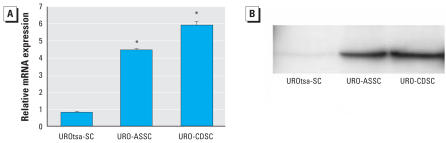
Expression of keratin 6a in parental UROtsa cells, UROtsa cells transformed by As^3+^, and UROtsa cells transformed by Cd^2+^ grown in serum-containing media. *(A*) Real-time RT-PCR analysis of keratin 6a mRNA levels. Relative mRNA levels were normalized to the change in *G3PDH* expression as described in “Materials and Methods” and reported as the mean ± SE. (*B*) Western blot analysis of keratin 6a protein levels. Gel bands developed chemiluminescently are shown. Statistically significant compared with parental UROtsa cells (*n* = 3 parallel cell culture samples).

Our laboratory performed a preliminary microarray analysis to screen for differences in mRNA expression between the parental UROtsa cells and the URO-CDSC- and URO-ASSC-transformed UROtsa cells that were grown in serum-containing growth medium. We performed this analysis by combining triplicate total RNA samples from each cell line and subjecting the total RNA to analysis using the U133 Plus 2.0 Affymetric array chip (Affymetrix, Inc.). The results of such an analysis are qualitative. This analysis suggested that the keratin 6a gene was overexpressed in the URO-ASSC cells. The first goal of this present study was to confirm the results of this preliminary microarray analysis. We prepared total RNA and cell lysates from confluent cultures of UROtsa, URO-CDSC, and URO-ASSC cells grown in serum-containing growth medium. We subjected the total RNA to real-time RT-PCR analysis to determine the expression of keratin 6a mRNA. Multiple parallel isolates (3–5) of each group of As^3+^- and Cd^2+^-transformed cells were available for analysis, and one isolate from each type of transfomation is shown as a representative of each group. The results of this analysis con-firmed that keratin 6a mRNA expression was significantly elevated in URO-ASSC cells compared with the UROtsa parental controls (Figure 1A). The results also showed that keratin 6a mRNA was similarly elevated in the CDSC cells compared with the UROtsa control cells (Figure 1A). There was no difference in keratin 6a mRNA expression between the transformed cell lines. The level of keratin 6a expression was determined relative to the expression of the *G3PDH* housekeeping gene It was also demonstrated using Western blot analysis that the keratin 6a protein (56 kDa) was elevated in both the URO-CDSC and URO-ASSC cells compared with the parental UROtsa cells, with the URO-CDSC cells exhibiting 39.4 ± 0.1-fold and the URO-ASSC cells exhibiting 24.2 ± 0.3-fold expression over control cells as determined by densitometry (Figure 1B). These findings con-firmed the preliminary microarray results for the URO-ASSC cells and extended the preliminary findings to also include the CDSC cells.

### Keratin 6a mRNA and protein in tumor heterotransplants produced by transformed UROtsa cell lines

**Figure 2:**
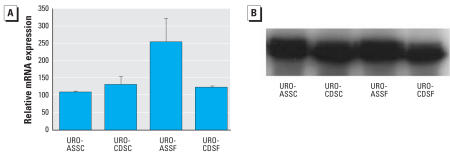
Expression of keratin 6a mRNA and protein in tumor heterotransplants produced by the URO-ASSC, URO-CDSC, URO-ASSF, and URO-CDSF cell lines. (*A*) Real-time RT-PCR analysis of keratin 6a mRNA levels. Relative mRNA levels were normalized to the change in G3PDH expression as described in “Materials and Methods” (*n* = 3 parallel cell culture samples, reported as mean ± SE). (*B*) Western blot analysis of keratin 6a protein levels. Gel bands were developed chemiluminescently.

**Figure 3:**
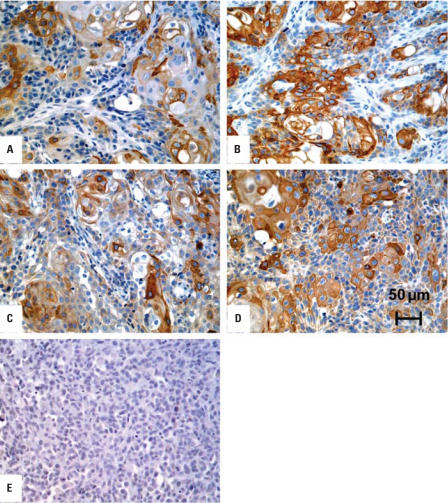
Immunohistochemical staining of keratin 6a in tumor heterotransplants produced by the URO-ASSC, URO-CDSC, URO-ASSF, and URO-CDSF cell lines. Keratin 6a staining in a heterotransplant resulting from the injection of URO-ASSF cells (*A*), URO-ASSC cells (*B*), URO-CDSF cells (*C*), and URO-CDSC cells (*D*). (*E* ) Absence of keratin 6a staining in heterotransplants derived from the human prostate carcinoma cell line PC-3. Scale bar, 50 μm.

We also prepared total RNA and protein lysates from subcutaneous tumor heterotransplants generated from the Cd^2+^- and As^3+^-transformed isolates that were originally generated from UROtsa cells grown in both serum-containing and serum-free growth medium. The results of this analysis disclosed that all four tumor heterotransplant groups expressed highly elevated levels of keratin 6a mRNA, 17- to 40-fold higher than the keratin 6a mRNA expression found in the two cell lines analyzed above (Figure 2A). A highly elevated level of keratin 6a protein was also demonstrated using Western blot analysis (Figure 2B). We used immunohistochemistry to determine the localization of keratin 6a expression in the cells of the tumor heterotransplants. This analysis showed that all four tumor heterotransplant groups contained very frequent profiles of cells that were positive for the presence of keratin 6a (Figure 3A–D). However, keratin 6a staining was focal, and all four tumors showed some areas of negative-staining tumor cells. In all cases, keratin 6a staining was diffusely cytoplasmic with no evidence of nuclear localization. Stroma within the tumors was negative for the expression of keratin 6a. All transformed isolates from each group gave similar results with focal staining and high levels of keratin 6a expression. As a control, heterotransplants derived from the human prostate carcinoma cell line PC-3 did not exhibit any staining for keratin 6a.

### Immunohistochemical staining of keratin 6a in archival specimens of human bladder cancer

**Figure 4:**
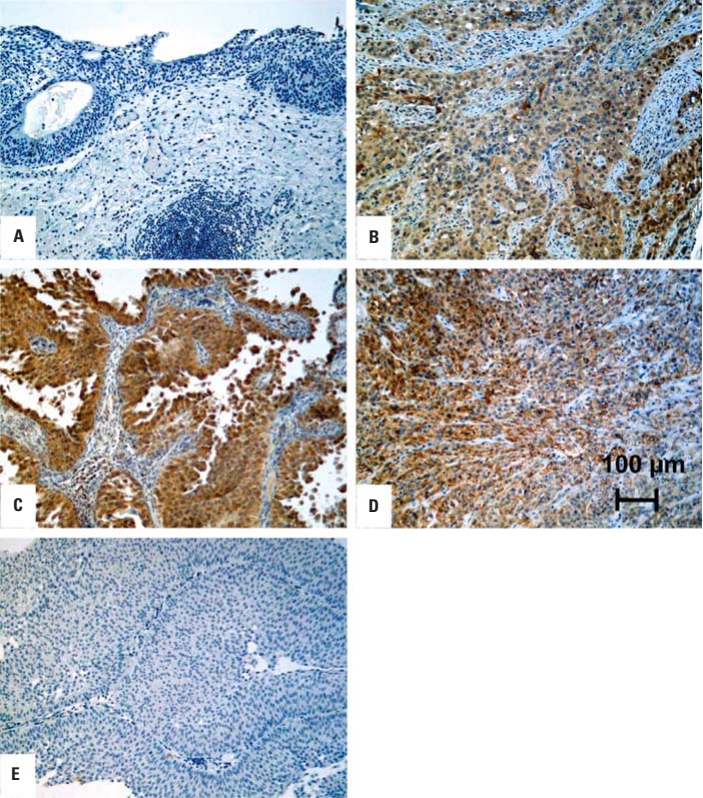
Immunohistochemical staining of keratin 6a in archival specimens of human bladder cancer. (*A*) Absence of keratin 6a staining in normal urothelium. (*B*) Staining of keratin 6A in a high-grade transitional cell carcinoma (TCC). (*C*) Staining of keratin 6a in a noninvasive high-grade TCC. (*D*) Staining of keratin 6a in an invasive high-grade TCC that has invaded the underlying muscle layer. (*E*) Absence of staining of keratin 6a staining in a low-grade urothelial cancer. Scale bar, 100 μm.

To determine if the findings in the UROtsa model system would translate to human bladder cancer, we performed keratin 6a staining on a small set of archival human bladder cancer specimens. An archival specimen that contained within a single tissue block both a profile of a high-grade urothelial cancer and adjacent normal (noncancerous) urothelium showed that the high-grade tumor was immunoreactive for keratin 6a, whereas the normal urothelium showed no staining for keratin 6a (Figure 4A, B). Two other specimens of high-grade cancer, one that had invaded the underlying muscle layer (Figure 4D) and one that had not (Figure 4C), were also shown to stain for keratin 6a. In all three high-grade tumors, staining was focal and cytoplasmic, with all the tumors showing areas that were both positive and negative for keratin 6a. A fourth archival specimen of a high-grade urothelial cancer was shown to have no staining for keratin 6a (data not shown), as was a fifth archival specimen of low-grade urothelial cancer (Figure 4E).

### Expression of keratin 6a in normal and transformed UROtsa cell lines grown in serum-free growth medium

**Figure 5:**
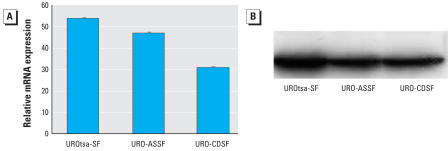
Expression of keratin 6a in parental UROtsa (UROtsa-SF), URO-CDSF, and URO-ASSF cell lines grown and maintained in serum-free growth medium. (*A*) Real-time RT-PCR analysis of keratin 6a mRNA levels. Relative mRNA levels were normalized to the change in *G3PDH* expression as described in “Materials and Methods” (*n* = 3 parallel cell culture samples, reported as mean ± SE). (*B*) Western blot analysis of keratin 6a protein levels. Gel bands were developed chemiluminescently.

To complete the analysis of keratin 6a expression in the UROtsa cell lines, we also determined the expression of keratin 6a mRNA and protein for the parental UROtsa cell line and the URO-CDSF- and URO-ASSF-transformed cell lines that had been grown and maintained in serum-free medium. The results of this determination unexpectedly showed that the parental UROtsa cells expressed high levels of both keratin 6a mRNA and protein compared with cells grown in serum-containing growth medium (Figures 1A, B, and 5A, B). The increase in expression of keratin 6a mRNA in the UROtsa cells grown in serum-free growth medium was approximately 50-fold higher than the level found for cells grown in serum-containing medium (Figures 1A and 5A) and approximately 140-fold in keratin 6a protein based on the optical density of the protein bands (Figures 1B and 5B). The expression of keratin 6a mRNA and protein was also significantly increased for the Cd^2+^- and As^3+^-transformed cell lines, but the increase was not as large as that found for the parental cell line with the levels of protein reaching only about 85-fold over the serum-containing control cells based on gel-band densitometry (Figure 5B).

**Figure 6:**
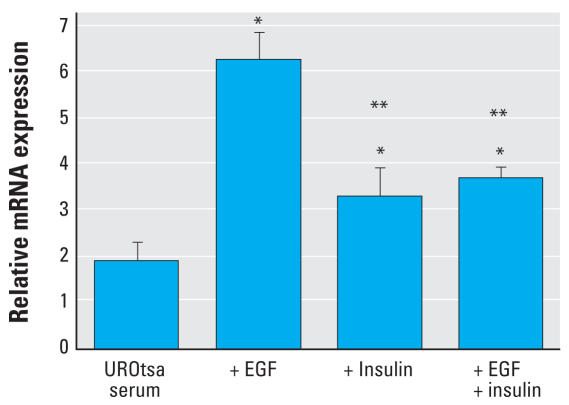
Effect of EGF and insulin on the expression levels of keratin 6a in UROtsa parental cells grown in serum-containing medium. +, presence. Confluent UROtsa cells were grown in serum-containing media with the addition of EGF alone or insulin alone or EGF + insulin for 16 hr. The expression of keratin 6a mRNA was determined using real-time RT-PCR. Relative mRNA levels were normalized to the change in *G3PDH* expression as described in “Materials and Methods” (*n* = 3 parallel cell culture samples, reported as mean ± SE). *Statistically significant compared with control cells grown in serum-containing media. **Statistically significant compared with UROtsa cells grown in serum-containing media with the addition of EGF.

**Figure 7:**
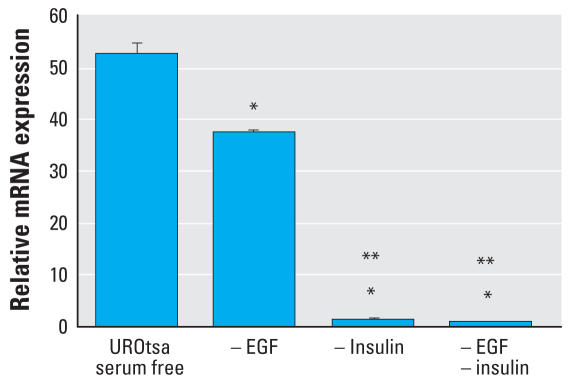
Effect of EGF and insulin deletion on the expression levels of keratin 6a in UROtsa cells grown in serum-free media. –, absence. Confluent UROtsa cells were cultured in serum-free media, or in serum-free media without EGF , or without insulin or without EGF and insulin for 16 hr. The RNA was extracted and analyzed by real-time RT-PCR for the expression of the keratin 6a gene. Relative mRNA levels were normalized to the change in *G3PDH* expression as described in “Materials and Methods” (*n* = 3 parallel cell culture samples, reported as mean ± SE). *Statistically significant compared with control cells grown in serum-free media. **Statistically significant compared with UROtsa cells grown in serum-free media without EGF.

Deletion and addition studies on the respective growth formulations were used to identify the factors in the growth medium that might be responsible for the increased expression of keratin 6a mRNA in parental UROtsa cells grown on serum-free growth medium. These studies showed that the addition of EGF (10 ng/mL) for 16 hr to confluent parental UROtsa cells grown in serum-containing growth medium resulted in a significant elevation in the expression of keratin 6a mRNA (Figure 6). We also observed significant increases in keratin 6a mRNA when insulin (5 μg/mL) or insulin + EGF (5 μg/mL + 10 ng/mL) was added to the serum-containing growth medium for 16 hr, although the increases were less than those noted for EGF alone (Figure 6). The addition of these components to the parental UROtsa cells grown in serum-containing growth medium had no adverse effect on the cells, as judged by light microscopy (data not shown). The deletion of EGF from the serum-free growth medium for 16 hr was shown to significantly reduce the expression of keratin 6a mRNA in the parental UROtsa cells (Figure 7). The deletion of insulin and insulin + EGF from the serum-free growth medium was also shown to significantly reduce the expression of keratin 6a mRNA in the parental UROtsa cells (Figure 7). The reduction of keratin 6a mRNA expression upon the deletion of insulin and insulin + EGF was greater than when only EGF was deleted from the serum-free medium. The deletion studies using the serum-free growth medium are more difficult to interpret than those on serum-containing medium, as the removal of any of the two components from the serum-free medium does have an adverse effect on the cells. At 16 hr postremoval of the growth factors, the cells are beginning to change morphology to a more rounded appearance. At 24 hr postremoval, the cells are rounded and beginning to detach from the culture dish. However, at 16 hr, cellular RNA is intact as monitored by agarose gel electrophoresis (data not shown). The removal or addition of growth factors had no effect on keratin 6a mRNA expression for the Cd^2+^- and As^3+^-transformed UROtsa cells (data not shown).

## Discussion

The first goal of this present study was to confirm a microarray analysis that suggested that keratin 6a might be overexpressed in urothelial cells transformed by As^3+^. This goal was achieved when it was shown that keratin 6a expression was increased in both Cd^2+^-and As^3+^-transformed UROtsa cells when compared with parental controls. The other goals of the study were similarly reached when it was shown the keratin 6a was expressed in tumor heterotransplants generated from the Cd^2+^- and As^3+^-transformed cells and that keratin 6a was overexpressed in some archival patient specimens of high-grade bladder cancer. The potential role that keratin 6a might play in the development and progression of bladder cancer is presently difficult to define because the keratin 6a gene has been only limitedly studied. The human gene was cloned and characterized by Takahashi and co-workers (1995) and shown to be one of several highly related type II keratin 6 iso-forms. Keratin 6a has been shown to be highly induced at the wound edge in epidermal keratinocytes that have been injured and to work in concert with keratin 16 in cytoskeletal reorganization during wound healing (Paladini et al. 1996). The analysis of the keratin 6a promoter has localized some of the critical regulatory sequences (Takahashi and Coulombe 1997), and keratin 6a knockout mice have been shown to have delayed wound healing (Wojcik et al. 2000). A mutation in keratin 6a has been shown to be involved in some cases of pachyonychia congenita types 1 and 2 (Garcia-Rio et al. 2005; Ward et al. 2003). Overall, keratin 6a and keratin 16 are associated with epidermal proliferation and wound repair (Safer et al. 2004; Stoler et al. 1988). A recent report associates keratin 6a expression with urothelial cell proliferation and wound repair (Sun 2006). Keratin 6a has been found to be overexpressed in the overlying epidermis of patients with dermatofibromas (Stoler et al. 1989), in keratinizing and nonkeratinizing squamous cell carcinomas of the cervix (Smedts et al. 1992), and in squamous cell carcinomas of the head and neck (Sesterhenn et al. 2005). To our knowledge keratin 6a has not been examined in human bladder cancer.

There are several possibilities as to how keratin 6a expression might affect the development and progression of bladder cancer. The first is that keratin 6a expression might be only a biomarker of upstream events in cancer development and progression and have no direct influence on either of these processes. Evidence for this hypothesis is based on the finding that keratin 6a expression in parental UROtsa cells is up-regulated by the presence of EGF and insulin. Additional evidence is provided by the observation that their Cd^2+^-and As^3+^-transformed counterparts are constitutively up-regulated for keratin 6a expression and are unaffected by the presence or absence of EGF and insulin in the growth medium. This finding is in agreement with another study that shows EGF was responsible for the induction of keratin 6a expression during the epidermal cells response to injury (Smiley et al. 2006). Furthermore, keratin 16, which is co-expressed with keratin 6a during epidermal proliferation, has been shown to be induced by EGF through cooperation with transcription factors Sp1 and c-Jun (Wang and Chang 2003). Our observation that insulin directly or indirectly through interaction with the insulin-like growth factor-1 receptor might also regulate keratin 6a expression to our laboratory’s knowledge has not been reported previously. Finally, an antibody that recognizes both keratins 5 and 6 has been used to identify squamous differentiation in breast carcinomas, and lesions so identified have been shown to have a very high frequency of EGF receptor expression (Bossuyt et al. 2005). Based on these scattered reports, one can hypothesize that keratin 6a overexpression in bladder cancer is a biomarker for malignant cells that have an activated growth factor pathway. Cells with an activated EGF signaling would be expected to have an enhanced potential to undergo progression. This is supported indirectly by the finding that keratin 6a expression is focal in tumor heterotransplants and in archival patient specimens. Thus, under this hypothesis, keratin 6a would be a biomarker for an activated cell proliferation pathway and need not have a direct role in cancer development or progression.

A second possibility, not exclusive of the one above, implicates keratin 6a in the process of urothelial cell transformation and progression that is driven by the environmental pollutant As^3+^. The association between arsenic exposure and urinary bladder cancer, typically transitional cell carcinoma, has been observed in the same endemic areas of the world where populations with skin cancer have been identified (Chiou et al. 1995; Luster and Simeonova 2004; Smith et al. 1998; Tsuda et al. 1995). In addition to neo-plasia, other more common manifestations of chronic arsenic exposure include hyperkeratosis and hyperpigmentation of the skin (Maloney 1996). Overall, there is substantial literature that shows skin to be unusually sensitive to both arsenic’s toxic and carcinogenic effects. One theory of why skin is highly responsive to arsenic exposure is that skin localizes and stores arsenic because of arsenic’s high keratin content and the corresponding favorable interaction with sulfhydryl groups (Kitchin 2001; Lingren et al. 1982). If high keratin content is indeed a driving force for As^3+^-induced epidermal keratinocyte carcino-genesis, then one can hypothesize that over-expression of keratin 6a in the bladder due to arsenite exposure would favor the accumulation of additional arsenic by the affected bladder urothelial cell. If keratin 6a was induced early during chronic arsenic exposure, this would drive the urothelial cell’s accumulation of intercellular arsenic metabolites and favor the progression of cell transformation. The accumulation of arsenic might be further enhanced by the co-expression of keratin 16. The finding that Cd^2 +^ also induced the expression of keratin 6a also fits well within this hypothesis, as Cd^2 +^ would also be expected to interact similarly with keratin.

The previous finding that tumor heterotransplants generated from Cd^2+^- and As^3+^-transformed UROtsa cells had increased squamous differentiation also correlates with the enhanced expression of keratin 6a (Sens et al. 2004). The association of keratin 6a and squamous differentiation is given further significance, as there is evidence that squamous differentiation in patients with bladder cancer is associated with a more aggressive cancer and a poor prognosis. Squamous differentiation has been shown to be an unfavorable prognostic feature in patients undergoing radical cystectomy, possibly because of its association with high-grade tumors (Frazier et al. 1993). Squamous differentiation has also been reported as predictive of a poor response in patients undergoing radiation therapy (Akdas and Turkeri 1991; Martin et al. 1989). In another report, squamous differentiation was associated with a poor response to systemic chemotherapy (Logothetis et al. 1989). Thus, evidence suggests that overexpression of keratin 6a could be a biomarker for tumor progression and that it might also participate in the enhanced urothelial cell accumulation of the environmental pollutant arsenic.

Keratins form a large gene family. It is now believed that there are as many as 37 keratin genes expressed in epithelial cells (Schweizer et al. 2006). The expression of these proteins has been studied extensively in cancer because of their use in aiding the differential diagnosis of neoplasms. Originally used to distinguish nonepithelial from epithelial tumors, keratin expression assessed immunohistochemically is now used to differentiate subclasses of epithelial tumors primarily for the determination of tumor in cases of morphologically undifferentiated tumors or in the identification of the origin of a metastatic tumor whose primary site is unknown (Chu et al. 2002). This is made possible because of the expression of the keratins being relatively tissue specific, with at least one member of the acidic low-molecular-weight keratin (class I) being co-expressed with at least one member of the basic, high-molecular-weight keratin (class II). Keratin expression is also dependent on the stage of development and the degree of terminal differentiation. In general, antibodies to keratins 7, 20, 14, and 5/6 are the most discriminatory with tumors derived from simple epithelia often positive for keratins 7 and/or 20, whereas the tumors are negative for keratins 14 and 5/6. The antibody to keratin 5/6 recognizes a common epitope in both keratins 5 and 6, but in most situations, it is detecting keratin 5 expression and not keratin 6. Tumors derived from stratified epithelia are often positive for keratins 14 and 5/6 and are negative for keratins 7 and/or 20 (Chu and Weiss 2002). Occasionally keratin expression changes, especially when there is a change in differentiation; some of these exceptions include some of the higher-grade and more malignant cancers. There are extensive studies detailing the rates of positivity among the different types of tumors, giving a good perspective on the likelihood in a specific diagnostic situation. Breast cancer is a good example where breast epithelia express keratins 7, 8, 18, and 19 but not keratin 20. The basal cells and myoepithelial layers are positive for keratins 5/6, 14, and 17 (Moll et al. 1983). Most breast tumors will be positive for keratin 7 but negative for keratins 5/6, 17, and 20 which is very useful for distinguishing breast adenocarcimas from adenocarcinomas from other glandular sites (Chu et al. 2000; Tot 1999). In poorly differentiated adenocarinomas from the breast, however, some may be positive for keratins 4, 5/6, 14, or 17, which is indicative of carcinomas from stratified epithelial sites. These tumors tend to have poor prognoses (Malzahn et al. 1998). Thus, it is not uncommon that keratin expression changes in the development of cancer, particularly when there is a change in differentiation. Bladder urothelium or transitional epithelium is a special class of epithelia with unique properties that allow for distention during bladder filling. It has keratin expression profiles that are similar to both simple and stratified epithelia in that keratins 7, 8, 18, and 19 (simple epithelial keratins) are expressed in all transitional layers, whereas basal cells express keratins 5/6, 13, and 17. Keratin 20 is expressed only in the upper layer known as the umbrella cells (Chu and Weiss 2002). Transitional cell carcinomas will most often express the stratified epithelial keratins keratins 5/6 and 17 as well as the simple epithelial keratins 7, 8, 19 and 20 (Achtstatter et al. 1988; Schaafsma et al. 1990). Those transitional cell carcinomas that exhibit squamous differentiation also express keratin 14 (Chu and Weiss 2001). Keratin 13 is more abundant in low-grade tumors than that of high grade tumors (Moll 1998). The expression of keratin 6a is unusual compared with other members of this gene family in that it is usually not expressed except during hyperproliferation. The skin is an excellent example where keratin 6a expression is transiently induced in keratinocytes at the leading edge of a wound. Thus, unlike other keratin isoforms, keratin 6a could potentially be a marker for transformation, at least for certain cancers such as transitional cell carcinomas. The present studies show that keratin 6a is induced in UROtsa cells malignantly transformed by Cd^2+^ or As^3+^ and that keratin 6a overexpression also occurs in high-grade human bladder cancer.
